# Liver abscess due to *Granulicatella adiacens* in an immunocompetent patient: Case report

**DOI:** 10.7705/biomedica.6504

**Published:** 2023-03-30

**Authors:** Julio García-Casallas, Katherine Patiño-Salazar, Eduardo Tuta-Quintero, Miguel Molina-Ardila

**Affiliations:** 1 Facultad de Medicina, Universidad de La Sabana, Chía, Colombia Universidad de la Sabana Universidad de La Sabana Chía Colombia; 2 Clínica Universidad de La Sabana, Chía, Colombia Universidad de la Sabana Universidad de La Sabana Chía Colombia

**Keywords:** Liver abscess, pyogenic, case reports, abscesos hepáticos piógenos, reporte de caso

## Abstract

Pyogenic liver abscesses due to Granulicatella adiacens are infections associated with high mortality, mainly in immunocompromised patients. The main microorganisms associated with liver abscesses are Klebsiella pneumoniae, and Escherichia coli, though it may also be polymicrobial. However, case reports describing liver infection by Granulicatella adiacens are scarce.

We present the case of an immunocompetent adult patient who presented 15 days of evolution consisting of quantified fever peaks associated with asthenia, adynamia, chills, jaundice and coluria. The initial clinical examination revealed a generalized icteric tint without abdominal pain, and blood pressure with a tendency to hypotension. Biliopancreatic confluent neoplasia, secondary cholangitis and sepsis of biliary origin were suspected, initiating fluid resuscitation and antibiotic therapy; blood cultures and complementary diagnostic studies were taken. Hepatobiliary ultrasound with evidence of an abscess of 73 x 62 mm in segment IV; the bile duct and pancreas were within normal limits. To better characterize the lesion evidenced in the liver, a contrast-enhanced computed tomography of the abdomen was performed. The patient completed antibiotic management with ciprofloxacin, vancomycin, and metronidazole in good condition and was successfully discharged.

This is the first pyogenic liver abscess reported caused by Granulicatella adiacens in an immunocompetent patient, in whom early microbiological diagnosis in conjunction with targeted antibiotic treatment and percutaneous drainage of the lesion was decisive in the clinical outcome.

## Resumen

Los abscesos hepáticos piógenos por *Granulicatella adiacens* son infecciones asociadas a una alta mortalidad, principalmente en pacientes inmunocomprometidos. Los principales microorganismos asociados a los abscesos hepáticos son *Klebsiella pneumoniae* y *Escherichia coli*, aunque pueden ser polimicrobianos. Sin embargo, los informes de casos que describen la infección hepática por *G. adiacens* son muy escasos.

Se presenta el caso de un paciente adulto inmunocompetente que presentó 15 días de evolución de picos febriles cuantificados asociados a astenia, adinamia, escalofríos, ictericia y coluria. El examen clínico inicial reveló un tinte ictérico generalizado sin dolor abdominal, y presión arterial con tendencia a la hipotensión. Se sospechó neoplasia biliopancreática confluente, colangitis secundaria y sepsis de origen biliar, y se inició reanimación con líquidos y antibioterapia. Se tomaron hemocultivos y estudios diagnósticos complementarios. En el ultrasonido hepatobiliar, se observó un absceso de 73 x 62 mm en el segmento IV; la vía biliar y el páncreas se encontraron dentro de los límites normales. Se realizaron múltiples pruebas moleculares de detección de microorganismos (FilmArray), y se identificó a *G. adiacens* como el principal agente patógeno. El paciente completó el manejo antibiótico con ciprofloxacina, vancomicina y metronidazol en buenas condiciones y fue dado de alta con éxito.

Este es el primer absceso hepático piógeno reportado causado por *G. adiacens* en un paciente inmunocompetente, en quien el diagnóstico microbiológico temprano en conjunto con el tratamiento antibiótico dirigido y el drenaje percutáneo de la lesión fueron determinantes en el resultado clínico.

Pyogenic liver abscesses are suppurative infections of the liver parenchyma associated with a mortality of 10 to 31% in the first 30 days of hospitalization, mainly in immunocompromised patients ^1^. The most common causes of pyogenic liver abscesses are abdominal infections such as appendicitis or peritonitis, bacteremia, bile duct infection and trauma, when no cause or risk factor associated with the abscess is found, it is described as cryptogenic ^1^. The main microorganisms associated with liver abscesses are *Klebsiella pneumoniae, Streptococcus milleri, Escherichia coli, Burkholderia pseudomallei* and *Staphylococcus aureus*^2^^,^^3^.

*Granulicatella adiacens* is a nutritional variant of *Streptococcus* from the *viridans* group, belonging more specifically to the genus *Abiotrophia* and in recent years described in the genus *Granulicatella* spp. ^2^. *Granulicatella adiacens* is present in the oral, gastrointestinal, and urogenital flora as commensal bacteria ^1^^,^^2^. At present, the main reported cases of infection by this bacterium are in patients diagnosed with endocarditis, septic arthritis, and bacteremia, mainly in users of breast implants, pacemakers, catheters and dental procedures ^3^. However, reports describing liver infection by this bacterium are scarce ^2^^,^^4^^,^^5^. We present the case of an immunocompetent adult patient who developed liver abscesses and sepsis due to infection by *G. adiacens*.

## Clinical case

The case of a 69-year-old male with a medical history of heavy smoking and allergy to penicillin due to rash and generalized itching after administration of an intramuscular dose is presented. The patient presented 15 days of evolution consisting of quantified fever peaks associated with asthenia, adynamia, chills, jaundice and coluria. The initial clinical examination revealed a generalized icteric tint without abdominal pain and blood pressure with a tendency to hypotension.

Admission laboratory exams reported leukocytosis at the expense of neutrophilia, thrombocytopenia, direct hyperbilirubinemia and compensated metabolic acidosis with hyperlactatemia (table 1), calculating a sequential organ failure assessment of 7 points. Therefore, biliopancreatic confluent neoplasia, secondary cholangitis and sepsis of biliary origin were suspected, initiating fluid resuscitation and antibiotic therapy with ciprofloxacin and metronidazole. Blood cultures and complementary diagnostic studies were performed simultaneously. The patient was transferred to the intermediate care unit, without requiring vasopressor support or invasive mechanical ventilation.

**Table 1:** Laboratory studies on admission to the emergency service

Leukocytes (cells/ml)	21,720
Neutrophils (cells/ml)	19,630
Hemoglobin (g/dl)	11.2
Hematocrit (%)	33
Platelets (per μΙ)	254,000
Albumin (g/L)	3.75
C-reactive protein (mg/L)	180
Alanine aminotransferase (U/L)	19
Aspartate aminotransferase	(U/L)
Bilirubin total (mg/dl)	2.25
Creatinine (mg/dl)	1.2
Ureic nitrogen (mg/dl)	17.6
Glucose (mg/dl)	120
Sodium (mEq/dl)	138

**Figure 1:**
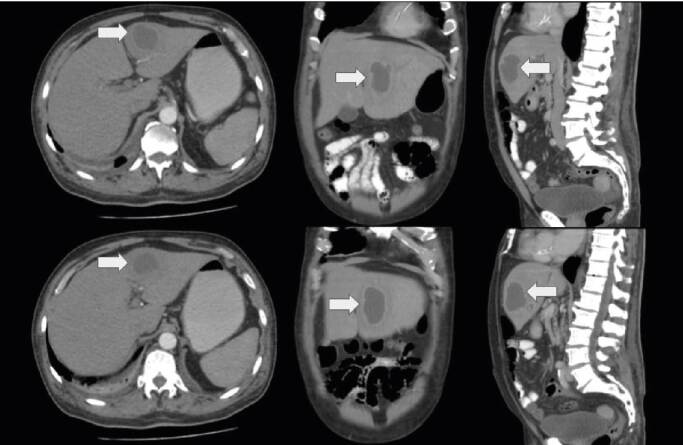
Computed tomography of abdomen and pelvis with intravenous contrast. The arrow shows a variable density internal pattern of a hypodense lesion in relation to the liver parenchyma, compatible with an abscess in the left hepatic lobe.

Hepatobiliary ultrasound showed an abscess of 73 x 62 mm in segment IV; the bile duct and pancreas were within normal limits. To better characterize the lesion evidenced in the liver, a contrast-enhanced computed tomography of the abdomen was performed (figure 1). The patient was referred for percutaneous drainage of the abscess under ultrasound and fluoroscopic guidance which was done with minimal difficulty, and the drained material was cultured.

Culture reports showed preliminary results with gram-positive cocci, so vancomycin was added to the antibiotic treatment. In addition, multiple microorganism detection molecular tests (FilmArray) were performed, identifying G. adiacens as the main pathogen. Antibiotic susceptibility testing allowed ciprofloxacin, vancomycin, and metronidazole to continue for one month. The patient completed the established antibiotic management in good conditions and was successfully discharged.

### Ethical considerations

The patient was not involved in the development of the study, and data were analyzed anonymously and approved by the research ethics committee of *Clínica Universidad de La Sabana*. The results will be disseminated to the scientific community in academic writing.

## Discussion

In this clinical case, the report of the microbiological isolation of *G. adiacens* is presented in a patient with no pathological history or user of devices that facilitate its colonization. The patient presented hypotension, leukocytosis, positive cultures, and the tomography showed a liver abscess. Currently, more than 90% of abscesses are polymicrobial ^4^, while in our case only *G. adiacens* was detected as the main pathogen ^5^.

Pyogenic liver abscesses can have a biliary (40.1%), cryptogenic (26.2%) or portal vein (16.1%) infection route ^4^^,^^6^. In Colombia, the most frequent hepatic abscesses are due to *Escherichia coli, Streptococcus viridans, Staphylococcus epidermidis, Streptococcus* spp., *Staphylococcus aureus* and *Pseudomonas aeruginosa*^3^^,^^4^^,^^6^. However, in patients with immunosuppression due to HIV infection, chemotherapy and organ transplantation, abscesses may occur due to fungi or opportunistic germs ^3^. Causes of immunosuppression such as human immunodeficiency virus infection or consumption of immunosuppressive drugs were ruled out for the patient here described.

*Granulicatella adiacens* are nutritionally variant gram-positive streptococci that have high nutritional needs for l-cysteine or pyridoxal to support growth, the latter is commonly found in human blood in low amounts of between 20 and 45 μg/ml ^1^^,^^2^^,^^5^. Alberti, et al. ^7^, evaluated 132 isolates in blood cultures of bacteria with high nutritional levels of pyridoxal, including G. adiacens and G. elegans, to evaluate the antimicrobial susceptibility pattern. Thirty-three per cent of the isolates were susceptible and 14% resistant to penicillin, finding *G. adiacens* in a high number of susceptible isolates (38.9% versus 10.8%). In our case, the result of the antibiogram revealed that all isolates were sensitive to gentamicin, streptomycin and vancomycin. On the other hand, although the pattern of resistance to penicillin is low, the penicillin allergy described in the clinical history did not allow starting antibiotics with beta-lactams, so cyclic lipopeptides and rifampicin were used.

In pyogenic liver abscesses, targeted antibiotic therapy and percutaneous drainage greatly decrease the mortality rate from 70% to less than 10%; however, focusing on an antibiotic regime is a medical challenge because these infections are usually polymicrobial due to anaerobic bacteria and members of the gastrointestinal flora ^8^^-^^10^. The evidence of extravascular infections by *G. adiacens* is limited, being the most frequent anatomical location in joints, ocular orbit, and lung, among others, or after joint prosthetic procedures ^3^^,^^6^. This case is novel because due to *G. adiacens* in an immunocompetent patient is unusual in the medical context ^2^^,^^9^^,^^10^^,^^11^.

Ideally, the pyogenic liver abscesses should be drained for a microbiological diagnosis and removal of purulent material as a complement to antibiotic treatment, the drainage route of the abscess should preferably be guided percutaneous and include anaerobic identification ^12^^,^^13^. However, the percutaneous or surgical drainage route should be selected according to conditions such as accessibility to the anatomical location, number of abscesses, size and clinical condition of the patient ^13^^,^^14^.

One of the main limitations was the non-availability of interventional radiology, however, the patient presented an adequate clinical evolution after minimally invasive drainage. The follow-up from admission to the emergency service allowed a detailed description of the clinical case, in addition, it was reassessed in external consultations.

## Conclusion

This is the first pyogenic liver abscess reported caused by *G. adiacens* in an immunocompetent patient, where early microbiological diagnosis in conjunction with targeted antibiotic treatment and percutaneous drainage of the lesion was decisive in the clinical outcome. Even though a high percentage of patients with *G. adiacens* infection present multiple comorbidities and compromise of the immune system, clinical and paraclinical suspicion in immunocompetent patients without medical history should be considered when faced with an intra-abdominal focus of infection and pyogenic liver abscesses.
